# Document triage for identifying protein–protein interactions affected by mutations: a neural network ensemble approach

**DOI:** 10.1093/database/bay097

**Published:** 2018-09-19

**Authors:** Ling Luo, Zhihao Yang, Hongfei Lin, Jian Wang

**Affiliations:** College of Computer Science and Technology, Dalian University of Technology, Dalian, China

## Abstract

The precision medicine (PM) initiative promises to identify individualized treatment depending on a patients’ genetic profile and their related responses. In order to help health professionals and researchers in the PM endeavor, BioCreative VI organized a PM Track to mine protein–protein interactions (PPI) affected by genetic mutations from the biomedical literature. In this paper, we present a neural network ensemble approach to identify relevant articles describing PPI affected by mutations. In this approach, several neural network models are used for document triage, and the ensemble performs better than any individual model. In the official runs, our best submission achieves an F-score of 69.04% in the BioCreative VI PM document triage task. After post-challenge analysis, to address the problem of the limited size of training set, a PPI pre-trained module is incorporated into our approach to further improve the performance. Finally, our best ensemble method achieves an F-score of 71.04% on the test set.

## Introduction

The precision medicine (PM) initiative promises to identify individualized treatment depending on a patients’ genetic profile and their related responses. In order to help health professionals and researchers in the PM endeavor, one goal is to leverage the knowledge available in the scientific published literature and extract clinically useful information that links genes, mutations and diseases to specialized treatments (1). Proteins and their interactions are the building blocks of metabolic and signaling pathways regulating cellular homeostasis (2). Therefore, understanding how allelic variation and genetic background influence the functionality of these pathways is crucial for predicting disease phenotypes and personalized therapeutical approaches.

Despite previous studies in protein–protein interaction (PPI) (3, 4) and mutation extraction (5), no one has investigated how to combine these efforts in order to help assessing and curating the clinical significance of genetic variants, an essential step toward PM. Thus, the PM task in BioCreative VI focuses on identifying and extracting from the biomedical literature PPIs affected by genetic mutations (PPIm) (6). This challenge consists of two subtasks. The first subtask is document triage that focuses on identifying relevant PubMed citations describing PPIm. The second subtask is relation extraction. Participants in this task will be expected to build automated methods that are capable of extracting experimentally verified PPIm. For the challenge, we participated in the first subtask (document triage).

Automatic document triage is a fundamental step for text mining and has received much attention. In the previous works, the state-of-the-art traditional machine learning methods [such as support vector machine (7), Naive Bayes (8) and maximum entropy (9)] depend on effective feature engineering, which is still a labor-intensive and skill-dependent task. Recently, deep learning methods, which are representation learning methods that compose simple but non-linear modules to obtain multiple levels of representation (10), have become prevalent in the machine learning research community. For the document triage task, several neural network-based deep learning methods [e.g. convolutional neural network (CNN) (11), recurrent neural network (RNN) (12) and combination of them (13)] have been proposed and exhibit promising results. Furthermore, above-mentioned methods have also been used to classify the biomedical literature, e.g. classifying PPI articles (14, 15) and prioritizing Comparative Toxicogenomics Database-relevant articles (16, 17). Compared with the traditional machine learning methods, the key advantage of deep learning methods is that these layers of features are not designed by human engineers, and therefore, the least feature engineering is needed. However, these deep learning methods generally require a large collection of manually labeled examples while creating one is time consuming and expensive, especially in the biomedical domain.

In this paper, we propose a neural network ensemble approach for the BioCreative VI PM document triage task. In this approach, five individual neural network models [i.e. LSTM (long-short term memory), CNN, LSTM-CNN, recurrent CNN (RCNN) and hierarchical LSTM (HieLSTM)] are used for document triage. To address the problem of the limited size of training set, a PPI pre-trained module with the existing labeled PPI corpora is incorporated into each neural network model. Afterwards, the ensemble model is built by combining five models’ results with three different alternatives (i.e. majority voting, weighted majority voting and a logistic regression classification) to further improve the performance. In addition, we explored the effect of additional features [such as part of speech (POS) and named entity recognition (NER) features] for the neural network models in the PM document triage task. Experimental results show that our approach achieves a better performance (an F-score of 71.04%) than the method of the team ranking first (an F-score of 69.06%) in the BioCreative VI PM task.

In the rest of this paper, first, our approach is described in detail, including the features, five individual neural network models, the PPI pre-trained module and the methods of model ensemble. Then, the experimental results are presented and discussed. Finally, some concluding remarks and directions for further research are offered.

## Materials and methods

**Figure 1:**
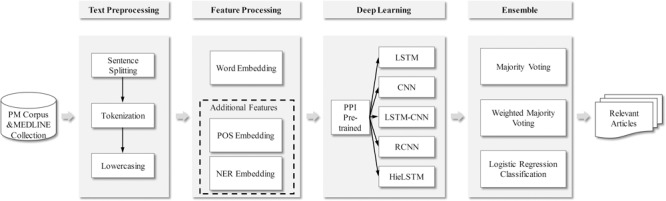
The processing flow of our method.

In this section, our approach for the BioCreative VI PM document triage is described. The processing flow of our method is shown in Figure 1. Firstly, some preprocessing steps including text sentence splitting, tokenization and lowercasing are performed. Secondly, a word embedding is learned with large amounts of unlabeled data with the fastText tool (18). Moreover, the additional features (i.e. POS and NER embeddings) are introduced into the model. Then with the embeddings as input, five neural network models are trained by the PM training set. Additionally, a PPI pre-trained module with the existing labeled PPI corpora is incorporated into each neural network model. Finally, the results from these models are combined by three different alternatives (i.e. majority voting, weighted majority voting and a logistic regression classification). The process is described in details in the following sections.

### Text preprocessing

First, article titles and abstracts are extracted from the data set. The extracted text is then split into the sentences, tokenized using the Stanford CoreNLP tool (19) and converted to lowercase. Note that the tokenization of the Stanford CoreNLP tool does not split text into segments at the dash (−) character. However, in the biomedical articles, two protein entity names are always combined into one token using dash character to express their interaction, such as ‘Utp6–Utp21 interaction’, ‘CheA–CheY binding interactions’ and ‘CHD7–CHD8’. To recognize the proteins in the PPI, we broke the text into separated segments at the dash character (e.g. ‘Utp6–Utp21’ is split into three tokens: ‘Utp6’, ‘–’ and ‘Utp21’). The processing can slightly improve the performance of our model.

### Features

Distributed word embedding is currently widely used in the field of natural language processing (NLP), especially based on the deep learning methods (20). The word embedding is used as the basic feature in our models. Moreover, to investigate the effects of traditional features (such as POS and named NER features), these features are also added into the models as additional features. All embeddings are parameters of the neural network model, and they can be optimized when the model is trained. Details of each of features are presented as follows.

### Word embedding

Word embedding, also known as distributed word representation, can capture both the semantic and syntactic information of words from a large unlabeled corpus and has attracted considerable attention from many researchers (20). Compared with the bag-of-words representation, word embedding is low dimensional and dense. In recent years, several tools, such as word2vec (21) and fastText (18), have been widely used in the field of NLP. To achieve a high-quality word embedding, we downloaded a total of 1 322 107 MEDLINE abstracts from the PubMed website with the query string ‘protein’ as the unlabeled data. Then the data and the PPI and PM corpora (a total of 25 134 abstracts and the details are described in the section ‘Experimental data sets and settings’) provided in the related BioCreative document triage tasks were used to train word embedding by the fastText tool as pre-trained word embedding.

### Additional features

Because of the complexity of the natural language and the specialty of the biomedical domain, some linguistic and domain resource features are often employed in traditional machine learning methods for the biomedical document triage (15, 22). We also explored the effect of the additional features (i.e. POS and NER features) for our neural network models. The POS information of each token was generated by the Stanford CoreNLP tool (19). In addition, NER tags (such as gene, chemical, disease and mutation entities) generated by the PubTator tagger (23) were also used as a feature. The NER feature of each token was encoded in BIO (Begin, Inside, Outside) tagging scheme, such as ‘CHD8\B-gene interacts\O with\O CHD7\B-gene,\O a\O protein\O which\O is\O mutated\O in\O CHARGE\B-disease syndrome\I-disease.\O’. In our experiments, the dimensions of the POS and NER embeddings are both five and they were initialized randomly.

### Description of the models

**Figure 2:**
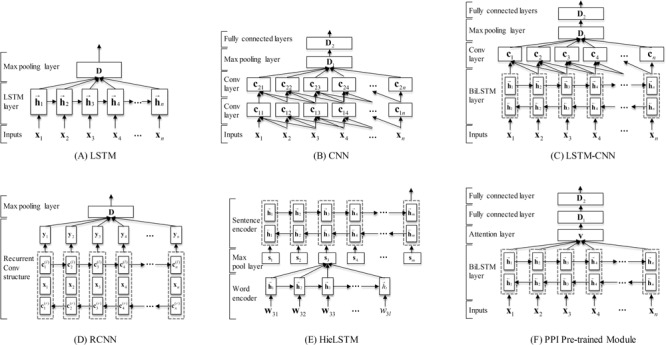
The neural network architectures of our models. **(A)** The LSTM model. **(B)** The CNN model. **(C)** The LSTM-CNN model. **(D)** The RCNN model. **(E)** The HieLSTM model. **(F)** The PPI pre-trained module.

In this section, we describe in details the five individual neural network models (i.e. LSTM, CNN, LSTM-CNN, RCNN and HieLSTM) used in our ensemble and a PPI pre-trained module. The architectures of all models are illustrated in Figure 2.

### LSTM

An RNN model, namely LSTM (24), is designed by incorporating a memory cell with the gating mechanism to enable learning of long-range dependencies. Given a document, the model predicts the label probability of the document. The architecture of the LSTM model is illustrated in Figure 2A. Firstly, through feature processing the document is represented as a sequence of vectors **X** = [**x**_1_,…,**x***_t_*,…,**x***_n_*] where *n* is the length of the document. Next, the vectors are given as input to an LSTM layer. For the *t-*th word in the document, the LSTM layer takes as input **x***_t_* and produces the hidden state **h***_t_* based on the following formulas:

(1) \begin{equation*} {\mathbf{i}}_t=\sigma\!\!\left({\mathbf{W}}^{(i)}{\mathbf{x}}_t+{\mathbf{U}}^{(i)}{\mathbf{h}}_{t-1}+{\mathbf{b}}^{(i)}\right) \end{equation*}

(2) \begin{equation*} {\mathbf{f}}_t=\sigma\!\!\left({\mathbf{W}}^{(\,f)}{\mathbf{x}}_t+{\mathbf{U}}^{(\,f)}{\mathbf{h}}_{t-1}+{\mathbf{b}}^{(\,f)}\right) \end{equation*}

(3) \begin{equation*} {\mathbf{c}}_t={\mathbf{f}}_t\ast {\mathbf{c}}_{t-1}+{\mathbf{i}}_t\ast \tanh\! \left({\mathbf{W}}^{(c)}{\mathbf{x}}_t+{\mathbf{U}}^{(c)}{\mathbf{h}}_{t-1}+{\mathbf{b}}^{(c)}\right) \end{equation*}

(4) \begin{equation*} {\mathbf{o}}_t=\sigma\!\!\left({\mathbf{W}}^{(o)}{\mathbf{x}}_t+{\mathbf{U}}^{(o)}{\mathbf{h}}_{t-1}+{\mathbf{V}}^{(o)}{\mathbf{c}}_t+{\mathbf{b}}^{(o)}\right) \end{equation*}

(5) \begin{equation*} {\mathbf{h}}_t={\mathbf{o}}_t\ast \tanh \left({\mathbf{c}}_t\right) \end{equation*}

where $\sigma$ is the element-wise sigmoid function and $^\ast$ is the element-wise product. {**W**^(.)^, **U**^(.)^, **V**^(.)^} is the weight matrix set. {**b**^(.)^} is the bias vector set.

Then, the sequence of vectors **h***_1:n_* output from all LSTM cells at each time step are combined into a single vector **D** by a max pooling layer. The vector could be considered as the semantic representation of the document. At last, a classification layer with softmax function is used on this document vector to compute the predictive probabilities of the document types.

### CNN

In the CNN model, a convolution operation is applied to produce local features. The architecture of the CNN model is illustrated in Figure 2B. Given an input sequence **X** = [**x**_1_,…,**x***_t_*,…,**x***_n_*], a fixed size *k* window approach is used to capture each element’s context information. Then, a matrix operation, as shown in formula (6), is applied to each successive window in the sequence:

(6) \begin{equation*} \mathbf{C}=\mathrm{ReLU}\!\left({\mathbf{W}}^{(con)}\bullet {\mathbf{X}}_{t:t+k-1}+{\mathbf{b}}^{(con)}\right) \end{equation*}

where **W**^(*con*)^ is the transformation matrix that is the same across all windows in the document and **b**^(*con*)^ is the bias vector. ReLU is the rectified linear unit function (25), and **C** is the convolutional layer result.

In our CNN model, two consecutive small convolutional layers (window size, *k* = 3) are stacked to extract convolutional features. For the document, one convolution operation extracts *n*-gram features over tokens where *n* is window size 3. Two consecutive convolutional layers can be in fact seen as how to best combine these different 3-gram features. Afterwards, a max pooling layer is used to extract global features from the convolutional layer. The next layer is a fully connected layer to an output layer with a softmax function.

### LSTM-CNN

In general, CNN is capable of extracting local information, and LSTM can capture long-range dependencies. So we combined the two neural network architectures into a model, namely LSTM-CNN. The architecture of the LSTM-CNN model is illustrated in Figure 2C. The intuition behind this model is that the LSTM layer can firstly capture the global context for each token and then the CNN layer can further extracts *n*-gram features over the tokens. The model mainly consists of two parts: a bidirectional LSTM (BiLSTM) layer and a convolution layer. Firstly, a document is represented as a sequence of embeddings. Next, the embeddings are given as input to a BiLSTM layer. In the BiLSTM layer, a forward LSTM computes a representation of the sequence from left to right, and another backward LSTM computes a representation of the same sequence in reverse. These two distinct networks use different parameters, and then the representation of a word is obtained by concatenating its left and right context representations. Then, a tanh (i.e. the hyperbolic tangent) function on top of the BiLSTM is used to learn higher features. Next, the features are fed into a convolution layer as shown in formula (6), and a max pooling layer is used to extract global features from the convolution layer. Afterwards, the features are fed into a fully connected layer. Finally, a classification layer with a softmax function is used to predict the probabilities of the document types.

### RCNN

Similar with the LSTM-CNN model, Lai *et al*. (13) proposed an RCNN for document classification. In the model, the recurrent structure is applied to capture the contextual information as much as possible when learning word representations of documents, which may introduce considerably less noise compared to a conventional window-based neural network. Moreover, the model can reserve a large range of the word ordering when learning representations of articles.

The architecture of our RCNN model is illustrated in Figure 2D. In our implementation of the RCNN model, a BiLSTM is used to capture the contexts. We define ${\mathbf{c}}_t^{(l)}$ as the left context of *t-*th word and ${\mathbf{c}}_t^{(r)}$ as the right context of *t-*th word in the document. The vector **x***_t_* is the word embedding of the *t-*th word. Then the left context ${\mathbf{c}}_t^{(l)}$ and the right context ${\mathbf{c}}_t^{(r)}$ are calculated using the following formulas:

(7) \begin{equation*} {\mathbf{c}}_t^{(\,l\,)}={\mathrm{LSTM}}_l\left({\mathbf{x}}_{t-1}\right) \end{equation*}

(8) \begin{equation*} {\mathbf{c}}_t^{(r)}={\mathrm{LSTM}}_r\left({\mathbf{x}}_{t+1}\right) \end{equation*}

where LSTM*_l_* is a forward LSTM computes a representation of the sequence from left to right and LSTM*_r_* is another backward LSTM computes a representation of the sequence in reverse. Then, the final representation of word ${\mathbf{w}}_t=\left[{\mathbf{c}}_t^{(\,l\,)};{\mathbf{x}}_t;{\mathbf{c}}_t^{(r)}\right]$ is the concatenation of the left context vector ${\mathbf{c}}_t^{(\,l\,)}$, the word embedding **x***_t_* and the right context vector ${\mathbf{c}}_t^{(r)}$. After the representation of word **w***_t_* is obtained, a tanh activation function is applied and the result **y***_t_* is fed into the next layer. Finally, a max pooling layer is also employed that automatically judges which features in the document play key roles, and a softmax function is used to classify documents.

### HieLSTM

Recently, Yang *et al.* (26) proposed a hierarchical attention network (HAN) for document classification. The model is designed to capture two basic insights about document structure. First, since documents have a hierarchical structure (words form sentences, sentences form a document), a document representation is constructed by first building representations of sentences and then those are aggregated into a document representation. Second, it is observed that different words and sentences in a document are differentially informative.

Similar with HAN model, we developed a HieLSTM model that has a hierarchical structure of documents using LSTMs. The architecture of our HieLSTM model is illustrated in Figure 2E. The hierarchical model mainly consists of a word encoder and a sentence encoder. For the word encoder, it is the same as LSTM model descripted above. Given a sentence, firstly through feature processing the sentence is represented as a sequence of vectors **W** = [**w**_1_,…,**w***_t_*,…,**w***_l_*]. Next, the vectors are given as input to an LSTM layer. Then the sequence of vectors output from all LSTM cells at each time step are combined into a single vector **s***_t_* by the max pooling layer. The vector could be considered as the semantic representation of the sentence. For the sentence encoder, a document vector can be obtained in a similar way of the word encoder. After a sequence of sentence vectors **S** = [**s**_1_,…,**s***_t_*,…,**s***_m_*] is obtained by the word encoder, a BiLSTM layer is used to encode the sentences. Then the output of the BiLSTM layer at the last time step is used as the whole document representation to classify the document into different types.

### PPI pre-trained module

In practice, the performances of deep learning models often depend on the labeled training corpus scale. The model often achieves the better performance on the large corpus than the small one. In this challenge, the training set provided by the organizers of the BioCreative VI PM document triage task is not large and consists of 4082 annotated PubMed articles (title and abstract) (27). However, automatic PPI article classification has been addressed in the previous BioCreative challenges [i.e. BioCreative II (Protein Interaction Article Subtask1) (28), BioCreative II.5 (Article Classification Task) (29) and BioCreative III (Article Classification Task-BioCreative III) (4)]. In these challenges, the document triage task is a binary classification (true/false) of articles whether containing PPI annotations. These corpora consist of a total of 19 642 annotated PubMed articles (title and abstract). Although PPI described in these articles is not always PPIm, our intuition is that these PPI corpora are helpful to improve the model performance since the PPIm-relevant article should be the PPI-relevant article and the PPI-irrelevant article should be the PPIm-irrelevant article. With this assumption, we propose a PPI pre-trained module to improve the performances of our neural networks models.

For the PPI pre-trained module, an attention-based BiLSTM model is built to identify PPI-relevant articles. The architecture of the PPI pre-trained module is illustrated in Figure 2F. Concretely, given a document, through feature processing the document is represented as a sequence of vectors. Next, the vectors are given as input to a BiLSTM layer. Then the sequence of vectors **h***_1:n_* output from all LSTM cells at each time step are combined into a single vector **v** by an attention layer using the following formulas:

(9) \begin{equation*} {u}_i=\tanh\!\left({\mathbf{W}}^{(a)}{\mathbf{h}}_i+{b}^{(a)}\right) \end{equation*}

(10) \begin{equation*} {\alpha}_i=\frac{\exp\!\left({u}_i\right)}{\sum_i\exp\!\left({u}_i\right)} \end{equation*}

(11) \begin{equation*} \mathbf{v}=\sum \limits_i{\alpha}_i{\mathbf{h}}_i \end{equation*}

Afterwards, the features are fed into two consecutive fully connected layers. Finally, a classification layer with a softmax function is used to identify PPI-relevant articles.

**Figure 3:**
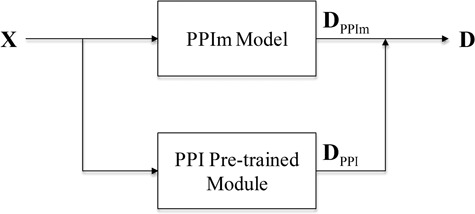
The architecture of our model with the PPI pre-trained module.

After the attention-based BiLSTM model is trained on the BioCreative PPI corpora, the model removing the last classification layer is used as the PPI pre-trained module to be incorporated into each individual neural network model (denoted as the PPIm model) described above. As shown in Figure 3, given a document, we define **D**_PPIm_ as the representation vector of the document learned by the PPIm model and **D**_PPI_ as the representation vector of the document learned by the PPI pre-trained module. Then, the final representation of the document **D** = [**D**_PPIm_; **D**_PPI_] is the concatenation of the two vectors.

### Model ensemble

In our experiments, three alternatives (majority voting, weighted majority voting and a logistic regression classification) are investigated to combine the results of the five individual models into an ensemble.

For the weighted voting method, we define the decision of the *t-*th classifier as ${d}_{t,j}\in \left\{0,1\right\}$. The ensemble result then chooses class *J* that receives the highest number of votes:

(12) \begin{equation*} \sum \limits_{t=1}^T{w}_t{d}_{t,J}=\underset{j=1}{\overset{C}{\max }}\sum \limits_{t=1}^T{w}_t{d}_{t,j} \end{equation*}

where *w_t_* is the weight of classifier *t*, ∑*w_t_* = 1 and *d_t,j_* is 1 or 0 depending on whether classifier *t* chooses *j* or not. We found the best setting of weights via brute force grid search, quantizing the coefficient values in the interval [0, 1] at increments of 0.1. The search was evaluated on our development set to avoid overfitting.

When the weights of all classifiers are set to 1 in the formula (12), the weighted majority voting becomes the majority voting. The ensemble result chooses class *J* that receives the highest number of votes. In our experiments, if the positive class and the negative class receive the same number of votes, the ensemble result chooses the negative class.

For a logistic regression classification method, the ensemble result is produced using a logistic regression model to estimate the conditional probability that an instance **x***_i_* belongs to a specific class *y_i_* as follows:

(13) \begin{equation*} P\left({y}_i|{\mathbf{x}}_i\right)=\frac{\exp\! \left(\sum \limits_j{\theta}_j{x}_{ij}\right)}{1+\exp\! \left(\sum \limits_j{\theta}_j{x}_{ij}\right)} \end{equation*}

where *x_ij_* is the representation of *j-*th feature of *i-*th instance and θ is the parameter. If there are *N* individual models, 2^*^N features are produced (two features per model) with the *P*-values of the negative and positive classes predicted by the respective model. Then, the features as inputs are fed into the logistic regression classifier to obtain the final result.

## Results and discussion

In this section, first the experimental data sets and settings are introduced, and then the experimental results and discussion are presented.

### Experimental data sets and settings

The organizers of the BioCreative VI PM document triage task provided a corpus (Data_PPIm_) including the training and test sets. The training set consists of 4082 annotated PubMed articles (title and abstract) as relevant or not relevant, and the test set consists of 1427 articles (http://www.biocreative.org/tasks/biocreative-vi/track-4/). In our experiments, we randomly selected the 10% of the training set as the development set to tune the hyperparameters and the models were evaluated on the test set (performances of individual models on our development set are also provided in Supplementary Material). For the PPI pre-trained module, we collected gold standard PPI corpora from previous BioCreative challenges, including BioCreative II, II.5 and III. Then all PPI corpora were combined into a PPI data set (Data_PPI_). Then, the 10% of the PPI data set was used as the development set to tune the hyperparameters, and the remaining data were used to train the PPI pre-trained model. Additionally, a total of 1 322 107 MEDLINE abstracts were downloaded from the PubMed website with the query string ‘protein’ as the unlabeled data. Then all the data were used to train word embedding by the fastText tool as pre-trained word embedding. Table 1 describes the statistics of various corpora. The document triage performance was measured with precision, recall and F-score (F1), which were calculated by the official evaluation scripts (https://github.com/ncbi-nlp/BC6PM).

**Table 1:** Statistics of various corpora used in our experiments

**Corpus Name**	**Positive**	**Negative**	**Total**
BioCreative VI PM training set	1729	2353	4028
BioCreative VI PM test set	704	723	1427
BioCreative II PPI corpus	3874	2298	6172
BioCreative II.5 PPI corpus	124	1066	1190
BioCreative III PPI corpus	2732	9548	12 280
PubMed unlabeled data			1 322 107

The parameters of our models in the word embedding are initialized with 50-dimensional pre-trained word embeddings (the performances of the higher dimensional word embeddings and the word embeddings trained by wor2vec tool were also tested, but no better performance was achieved. And the results are provided in Supplementary Material: ‘Performance of word embeddings’) and other parameters are initialized at random from a uniform distribution. Then, all parameters of models [except the CNN model using Adadelta (30)] are optimized using RMSprop (31) to minimize the categorical cross-entropy loss. Moreover, we tuned the hyperparameters on the development set by random search (32). The main hyperparameters of our models can be found in Supplementary Material: ‘Hyperparameter settings’. The number of epoch is chosen by early stopping strategy (33) on the development set. Our models were implemented using open-source deep learning library keras (https://keras.io) and trained on a NVIDIA Tesla K40 GPU.

### Performance of individual models and the effect of the PPI pre-trained module

Table 2 reports the results of individual models on the Data_PPIm_ test set, including the preliminary models and the models with the PPI pre-trained module described in the section ‘Description of the models’ (all models only used word embedding as inputs). For the models with the PPI pre-trained module, two variants are designed. ‘Model + PPI_pre_(Static)’: the model with the static PPI pre-trained module. After the PPI pre-trained module are trained on the Data_PPI_, all the parameters of the module are kept static and only the other parameters of the model are tuned on the Data_PPIm_. ‘Model + PPI_pre_(Tuned)’: the model with the tuned PPI pre-trained module. Same as above but all the parameters of the PPI pre-trained module are fine-tuned on the Data_PPIm_. Moreover, the McNemar’s significance test (34) was applied to compare the performance of the different models. And the results are provided in Supplementary Material: ‘Statistical analysis of significance’.

**Table 2:** Performance of individual models

**Model**	**Precision**	**Recall**	**F1**
LSTM	58.50	81.11	67.98
LSTM+PPI_pre_(Static)	58.42	85.35	69.36
LSTM+PPI_pre_(Tuned)	58.52	86.36	69.76
CNN	59.29	80.68	68.35
CNN + PPI_pre_(Static)	55.57	**90.63**	68.90
CNN + PPI_pre_(Tuned)	59.35	86.08	70.26
LSTM-CNN	57.24	85.37	68.53
LSTM-CNN + PPI_pre_(Static)	57.17	88.35	69.42
LSTM-CNN + PPI_pre_(Tuned)	**62.09**	80.97	**70.28**
RCNN	61.36	78.27	68.79
RCNN+PPI_pre_(Static)	58.14	86.22	69.45
RCNN+PPI_pre_(Tuned)	57.38	88.92	69.75
HieLSTM	57.74	82.10	67.80
HieLSTM+PPI_pre_(Static)	56.55	87.07	68.57
HieLSTM+PPI_pre_(Tuned)	57.69	87.36	69.49

Note: The boldfaced numerals are the highest values in the corresponding column.

The results show that, among the preliminary models, RCNN achieves the highest F-score of 68.79%, while CNN and LSTM-CNN perform slightly better than LSTM and HieLSTM. Additionally, we found that the combination of CNN and LSTM (i.e. RCNN and LSTM-CNN) performs better than any individual of them. When the PPI pre-trained module is added into the preliminary models, all models achieve performance improvements. The LSTM-CNN with PPI_pre_(Tuned) achieves the highest F-score of 70.28%. It demonstrates that the prior PPI information can help boost the performance of the model for identifying PPIm. Compared with PPI_pre_(Static), higher F-scores (average F-score improvements of 1.62 vs 0.85% over the preliminary ones across various models) are achieved when PPI_pre_(Tuned) is added. The possible reason is that PPI_pre_(Static) can learn the PPI task-specific information, while PPI_pre_(Tune) can learn the PPI and PPIm task-shared feature by fine-tuning on the Data_PPIm_ training set.

To further explore the effectiveness of our proposed PPI pre-trained module, the LSTM model is chosen as a baseline, and the results of several comparisons are provided in Table 3. As can be seen from Table 3, only PPI_pre_ (i.e. the model proposed in the section ‘PPI pre-trained module’) achieves a high recall and a low precision, which shows the prior PPI information can find both most of PPIm-relevant articles and other noisy articles. When the Data_PPI_ and Data_PPIm_ training sets are simply combined to train the LSTM model, the model performance is not improved (67.68 vs 67.98% in F-score), while the LSTM (Pre-trained) model achieves the improvement (0.91% in F-score). The reason is that the size of training data is expanded but the noise is introduced into the data set as well (e.g. the positive instance in the Data_PPI_ may be the negative one in the Data_PPIm_) when the two training sets are simply combined. Unlike the LSTM(Combined Data), LSTM(Pre-trained) is first pre-trained on the Data_PPI_, then the parameters of the model are fine-tuned on the Data_PPIm_ training set. Therefore, the above-mentioned noise is not introduced. When our PPI pre-trained module is added into the model, the F-score is improved significantly [improvements of 1.78 and 1.38% with PPI_pre_(Tuned) and PPI_pre_(Static), respectively]. Compared with the LSTM(Pre-trained), our LSTM+PPI_pre_ methods can learn PPI and PPIm features by two modules (i.e. the PPIm model and the PPI pre-trained module), while the LSTM(Pre-trained) mixed all PPI and PPIm features into one module. In summary, the Data_PPI_ is helpful for the PPIm document triage, and our PPI pre-trained method is effective.

**Table 3:** The effect of the PPI pre-trained module

**Model**	**Precision**	**Recall**	**F1**
LSTM	58.50	81.11	67.98
LSTM+PPI_pre_(Static)	58.42	85.35	69.36
LSTM+PPI_pre_(Tuned)	58.52	86.36	**69.76**
LSTM(Combined Data)	63.51	72.44	67.68
LSTM(Pre-trained)	**63.80**	74.86	68.89
Only PPI_pre_	50.76	**95.03**	66.17

Note: ‘LSTM(Combined Data)’ denotes the Data_PPI_ and Data_PPIm_ training set are directly combined into a data set to train the LSTM model. ‘LSTM(Pre-trained)’ denotes the LSTM model is first pre-trained on the Data_PPI_, then the parameters of the model are fine-tuned on the Data_PPIm_ training set. ‘Only PPI_pre_’ denotes the result of only the PPI pre-trained module on the Data_PPIm_ test set.

### The effect of additional features on performance

We also investigated the effect of two additional features (i.e. POS and NER embeddings mentioned in the section ‘Additional features’) on the performances of the preliminary models and the models with PPI_pre_(Tuned). In our experiments, the concatenation of the word embedding and additional features as input is fed into the models. Moreover, Table 4 shows the results of these features’ different combinations.

**Table 4:** The effect of additional features on performance

	**Preliminary model**					**Model + PPI**_**pre**_ **(Tuned)**			
Model	Precision	Recall	F1	△		Precision	Recall	F1	△
LSTM	58.50	81.11	67.98			58.52	86.36	69.76	
+ POS	60.06	80.97	68.97	+0.99		57.95	89.06	70.21	+0.45
+ NER	59.15	83.10	69.11	+1.13		57.67	89.20	70.05	+0.29
+POS + NER	56.94	83.95	67.85	−0.13		58.37	87.22	69.93*	+0.17
CNN	59.29	80.68	68.35			59.35	86.08	70.26	
+ POS	56.07	85.94	67.86	−0.49		57.25	87.50	69.21	−1.05
+ NER	55.72	**88.49**	68.39	+0.04		59.86	82.39	69.34	−0.92
+NER + POS	58.23	78.84	66.99	−1.36		59.40	84.38	69.72	−0.54
LSTM-CNN	57.24	85.37	68.53			**62.09**	80.97	**70.28**	
+ POS	57.00	83.24	67.67	−0.86		61.22	80.97	69.72	−0.56
+ NER	59.29	80.26	68.20	−0.33		59.17	83.38	69.22	−1.06
+POS + NER	58.38	86.08	**69.58**	+1.05		56.98	**89.91**	69.75	−0.63
RCNN	**61.36**	78.27	68.79			57.38	88.92	69.75	
+ POS	56.85	87.78	69.01	+0.22		57.24	85.94	68.71	−1.04
+ NER	58.54	85.23	69.40	+0.61		58.75	84.38	69.27	−0.48
+POS + NER	56.58	**88.49**	69.03	+0.24		59.65	82.53	69.25	−0.50
HieLSTM	57.74	82.10	67.80			57.69	87.36	69.49	
+ POS	55.34	86.08	67.37	−0.43		56.88	88.64	69.29	−0.20
+ NER	57.06	85.51	68.45	+0.65		56.85	86.08	68.47	−1.02
+POS + NER	55.82	**88.49**	68.46	+0.66		59.21	84.94	69.78	+0.29

Note: 69.93* in second to last column is the high F-score achieved by the model with both POS and NER features.

For the preliminary models, the results are somewhat inconsistent when these additional features are added into the models. When only POS feature is added, LSTM and RCNN achieve a slight performance improvement (average improvements of 0.61% in F-score across various models), while CNN, LSTM-CNN and HieLSTM perform even worse (an average decrease of 0.59% in F-score). When only NER feature is added, all models except LSTM-CNN achieve a performance improvement (an average improvement of 0.61% in F-score). When both POS and NER features are added, LSTM-CNN, RCNN and HieLSTM achieve a slight performance improvement (an average improvement of 0.65% in F-score), while LSTM and CNN perform worse (the F-score decreases by an average of 0.75%). Among these models, LSTM-CNN with POS and NER features achieves the highest F-score of 69.58%. In summary, the information of POS and NER can help boost the performance of some models and, especially, the information of NER is more effective. However, noise may be introduced into the models by the errors of the POS and NER tools that leads to the decrease in performances of some models.

For the models with PPI_pre_(Tuned), when the additional features are added, most models (i.e. CNN, LSTM-CNN and RCNN) perform worse. The plausible reason is that our PPI pre-trained module is pre-trained without additional features and the addition of these features makes the model confusion. Moreover, the errors of the POS and NER tools introduce noise into the models.

### Performance of different model combinations

As described in the section ‘Model ensemble’, three alternatives (i.e. majority voting, weighted majority voting and a logistic regression classification) are used to combine the model results into an ensemble. To select the best ensemble, we investigated the effect of these ensemble methods on the performances of the models with PPI_pre_(Tuned). Moreover, the effect of the voted assembly of the preliminary models without the PPI module has been investigated during the challenge (35). Note that the results in this paper and those presented in the paper (35) are slightly different since the organizers updated the test set after the challenge.

As can be seen from Table 5, the ensemble of five models without additional features using a logistic regression classification achieves the highest F-score of 71.04% and outperforms any individual model [an improvement of 0.76% in F-score than the best single model (70.28%, bold in the penultimate column of Table 4)]. When the results of five models with additional features are combined, the best ensemble achieves a slight improvement (0.22% in F-score) than the best single model with additional features (69.93%, bold in the penultimate column of Table 4). The reason is that, according to Table 4, since the additional features do not contribute to the performance improvement of five models, their ensemble could not be expected to output a much better result. Finally, when the results of these 10 models (5 models with additional features and 5 models without the features) are combined, the ensemble result has no further improvement.

**Table 5:** Performance of different model combinations

**Model**	**Method**	**Precision**	**Recall**	**F1**
Models +PPI_pre_(Tuned)	Voting	58.57	86.36	69.80
	Weighted voting	59.23	87.07	70.50
	Logistic regression	62.94	81.53	**71.04**
Models +PPI_pre_(Tuned) + Addfea	Voting	58.99	86.22	70.05
	Weighted voting	59.23	85.65	70.03
	Logistic regression	61.89	80.97	70.15
All	Voting	59.02	85.94	69.98
	Weighted voting	57.65	**88.35**	69.77
	Logistic regression	**63.51**	78.13	70.06

Note: Models+PPI_pre_(Tuned) denotes five models with PPI_pre_(Tuned) using the word embedding as input; Models+PPI_pre_(Tuned) + Addfea denotes five models with PPI_pre_(Tuned) using the concatenation of word embedding and additional features as input; All denotes all the 10 models.

### Performance comparison with other related works

To further demonstrate the effectiveness of our approach, the performance comparison between the top three results (6) (as noted in previous section, these results and those presented in paper (6) are slightly different since the organizers updated the test set after the challenge.) in the BioCreative VI PM document triage task and ours is shown in Table 6. Note that the result of team 421 is the result of the ensemble of our five preliminary models using weighted majority voting (i.e. our best submission for the BioCreative VI challenge). The results show that our best single model with a PPI pre-trained module obtains a higher F-score (70.28% in F-score) than the best result submitted in the task (69.06% in F-score). Moreover, our best ensemble achieves the highest F-score of 71.04%.

**Table 6:** Performance comparison with other related works

**Method**	**Precision**	**Recall**	**F1**
Team 418	62.89	76.56	69.06
Team 421	60.73	79.97	69.04
Team 374	57.00	**87.36**	68.98
Ours (single model)	62.09	80.97	70.28
Ours (ensemble)	**62.94**	81.53	**71.04**

### Error analysis

In addition, we manually analyzed the cases in which our best ensemble fails to classify the PPIm articles correctly. The predictions confusion matrix is shown in Table 7, which shows that false positives account for most of the classification error.

**Table 7:** The confusion matrix of our best ensemble on the Data_PPIm_ test set

**Actual**	**Predicted**	
	True	False
True	574	130
False	338	385

When analyzing incorrectly classified PPIm cases, we observed that many articles containing strong PPIm indicators are falsely identified as positive instances. These articles have similar expressions with positives but are actually negatives. For example, the article with PMID (PubMed ID): 16865698 contains some strong positive keywords (such as ‘protein’, ‘mutant’ and ‘interact’) but does not describe PPI influenced by genetic mutations. Our model misclassified the article as a positive one since it contains these strong positive keywords. For false negatives, we found that such strong positive keywords are missing or the positive indicators do not appear in the training set while it is difficult to accurately classify PPIm when the expression is rare (even none) in the training set, although the article describes PPIm. For example, the article with PMID: 17412961 describes PPI influenced by genetic mutations, but the common positive keywords (such as ‘mutant’ and ‘mutagenesis’) are replaced with other words (such as ‘spliceosomal’ and ‘aberrant’) that rarely appear in the training set. Our model misclassified such positive instances as the negative ones.

To sum up, the main reason for the errors may be that our model only depends on lexical features that may not contain sufficient information to solve semantic ambiguities in some cases. Even though automatic learning of high-level features is one advantage of deep learning methods, it is difficult for them to automatically learn the deep linguistic knowledge from the raw articles. Therefore, incorporating the deep linguistic analysis (e.g. syntactic and semantic analysis) into our method might further enhance our model’s performance. It will be explored in our future work.

## Conclusions

In this paper, we present a neural network ensemble approach to automatically identify PPIm-relevant articles. In this approach, a new PPI pre-trained module is introduced to utilize the exiting PPI data. In addition, the effect of additional features for the neural network models in the PM document triage task is explored. The experimental results show that (i) our PPI pre-trained module is proved to be effective to improve the performances of the deep learning models on the limited labeled PPIm data set and (ii) our ensemble of the neural network models using a logistic regression classification can achieve a further improvement. Owing to these advantages, our ensemble achieves the state-of-the-art performance on the BioCreative VI PM corpus (71.04% in F-score).

Our ensemble approach exhibits promising results for the PPIm document triage task. However, the additional features cannot achieve an improvement for our ensemble approach in our experiments. In the future work, we will focus on the effective methods of incorporating the additional features to improve the performance of our ensemble and deep linguistic analysis (e.g. syntactic and semantic analysis) will be explored in our future work.

## Supplementary Material

Supplementary Data

## Notes

**Database URL:** https://github.com/lingluodlut/BioCreativeVI-PM-Track
