# Supplementary material for: Document triage for identifying protein–protein interactions affected by mutations: a neural network ensemble approach
Source: Database (Oxford). 2018 Sep 19;2018:bay097. doi: 10.1093/database/bay097 (PMC6147215; doi:10.1093/database/bay097)
Supplement: Supplementary Data [file bay097_supp.doc]

**Supplementary Materials**

**Supplementary Material: Performance of word embeddings**

To investigate the effect of word embeddings on performance, we tested different dimensional word embeddings (i.e., 50, 100 and 150 dimensions) produced by the fastText and word2vec tools on the preliminary CNN model. Table S1 shows the performances of the different word embeddings on our development set. For the CNN model, the best performances on F-score are achieved with 50-dimensional word embeddings. Compared with the word embedding trained by the word2vec tool, the word embedding trained by fastText tool achieves better performance on our CNN model. Therefore, we finally chose the 50-dimensional word embedding trained by the fastText tool in our experiments.

**Table S1.** The performances of the different word embeddings on our development set.

| **Tool** | **Dimension** | **Precision** | **Recall** | **F1** |
| --- | --- | --- | --- | --- |
| Word2vec | 50 | 64.90 | 82.82 | 72.78 |
|  | 100 | 59.74 | 84.66 | 70.05 |
|  | 150 | 63.18 | 85.28 | 72.59 |
| FastText | 50 | 64.38 | **86.50** | **73.82** |
|  | 100 | 61.40 | 85.89 | 71.61 |
|  | 150 | **65.15** | 79.14 | 71.47 |

**Supplementary Material:** **Hyper-parameter settings**

In addition, training neural network-based model requires determining appropriate hyper-parameters. Details of the main hyper-parameters are summarized in Table S2.

**Table S2.** The main hyper-parameters of our models

| **Model** | **Hyper-parameter** | **Value** |
| --- | --- | --- |
| Common | Word embedding dimension | 50 |
|  | POS embedding dimension | 5 |
|  | NER embedding dimension | 5 |
|  | Dropout | 0.2 |
|  | RMSprop learning rate | 0.001 |
|  | Mini batch size | 32 |
| LSTM | LSTM state size | 50 |
| CNN | Convolutional layer size | 150 |
|  | Fully connected layer size | 30 |
| LSTM-CNN | BiLSTM state size | 50 |
|  | Convolutional layer size | 60 |
|  | Fully connected layer size | 30 |
| RCNN | BiLSTM state size | 50 |
|  | TimeDistributed fully connected layer size | 150 |
| HieLSTM | Word-level LSTM state size | 50 |
|  | Sentence-level BiLSTM state size | 100 |
| PPI pre-trained module | BiLSTM state size | 50 |
|  | First fully connected layer size | 100 |
|  | Second fully connected layer size | 50 |

**Supplementary Material: Performance of individual models on our development set**

To explore the effectiveness of our proposed PPI pre-trained module, the results of individual models on our development set and official test set are shown in Table S3. There is not a notable difference in the results between the development set and the test set. The CNN and the LSTM-CNN with PPI_pre_(Tuned) achieve the highest F-scores on our development set (76.19%) and the official test set (70.28%), respectively. When the PPI pre-trained module is added into the preliminary models, all models achieve improvements on both the development and test sets. It demonstrates that the Data_PPI_ is helpful for the PPIm document triage and our PPI pre-trained method is effective.

**Table S3. Performance of individual models on our development set and official test set.**

|  | **Our development set** | | |  | **Official Test Set** | | |
| --- | --- | --- | --- | --- | --- | --- | --- |
| **Model** | **Precision** | **Recall** | **F1** |  | **Precision** | **Recall** | **F1** |
| LSTM | 61.32 | 91.41 | 73.40 |  | 58.50 | 81.11 | 67.98 |
| LSTM+PPI_pre_(Static) | 69.11 | 80.98 | 74.58 |  | 58.42 | 85.35 | 69.36 |
| LSTM+PPI_pre_(Tuned) | 63.68 | 91.41 | 75.06 |  | 58.52 | 86.36 | 69.76 |
| CNN | 64.38 | 86.50 | 73.82 |  | 59.29 | 80.68 | 68.35 |
| CNN+PPI_pre_(Static) | 64.55 | 87.12 | 74.15 |  | 55.57 | **90.63** | 68.90 |
| CNN+PPI_pre_(Tuned) | **70.10** | 83.44 | **76.19** |  | 59.35 | 86.08 | 70.26 |
| LSTM-CNN | 63.80 | 86.50 | 73.44 |  | 57.24 | 85.37 | 68.53 |
| LSTM-CNN+PPI_pre_(Static) | 69.11 | 80.98 | 74.58 |  | 57.17 | 88.35 | 69.42 |
| LSTM-CNN+PPI_pre_(Tuned) | 69.23 | 82.82 | 75.42 |  | **62.09** | 80.97 | **70.28** |
| RCNN | 63.84 | 87.73 | 73.90 |  | 61.36 | 78.27 | 68.79 |
| RCNN+PPI_pre_(Static) | 63.56 | **92.03** | 75.19 |  | 58.14 | 86.22 | 69.45 |
| RCNN+PPI_pre_(Tuned) | 65.18 | 89.57 | 75.45 |  | 57.38 | 88.92 | 69.75 |
| HieLSTM | 62.13 | 89.58 | 73.37 |  | 57.74 | 82.10 | 67.80 |
| HieLSTM+PPI_pre_(Static) | 64.44 | 88.96 | 74.74 |  | 56.55 | 87.07 | 68.57 |
| HieLSTM+PPI_pre_(Tuned) | 63.91 | 90.18 | 74.81 |  | 57.69 | 87.36 | 69.49 |

The bold values denote the highest values

**Supplementary Material: Statistical analysis of significance**

To investigate the impact of the difference on the performance variation of these different models, we employed the McNemar’s significance tests . Table S4 summarizes the $\chi$ statistic values for pairwise comparison on different models using the McNemar’s significance tests. The results than or equal to 3.84 are considered to be significant at the 0.05 level. Firstly, among the preliminary models, RCNN model achieves the best F-score and significantly differs from LSTM-CNN, LSTM and HieLSTM models at the 0.05 level. The other four models do not show statistically significant differences between each other. Secondly, when the PPI pre-trained module is added into the preliminary models, all models achieve improvements. The LSTM-CNN with PPI_pre_(Tuned) achieves the highest F-score of 70.28%. Among the models with the tuned PPI pre-trained module, LSTM-CNN model significantly differs from other four models. In addition, a statistically significant difference exits between CNN and RCNN models. Finally, compared with the corresponding preliminary models, RCNN, CNN and LSTM-CNN models with the tuned PPI pre-trained module achieve significant improvements. And the improvements have been proven to be significant using McNemar’s test.

**Table S4.** The $\chi$ statistic values for pairwise comparison on different models using the McNemar’s significance tests.

|  | **LSTM** | **LSTM^1^** | **LSTM^2^** | **CNN** | **CNN^1^** | **CNN^2^** | **LSTM**  **-CNN** | **LSTM**  **-CNN^1^** | **LSTM**  **-CNN^2^** | **RCNN** | **RCNN^1^** | **RCNN^2^** | **HieLSTM** | **HieLSTM^1^** | **HieLSTM^2^** |
| --- | --- | --- | --- | --- | --- | --- | --- | --- | --- | --- | --- | --- | --- | --- | --- |
| **LSTM** | - | 0.27 | 0.46 | 0.45 | **5.27** | 2.33 | 0.81 | 0.32 | **11.29** | **5.80** | 0.04 | 0.07 | 0.45 | 2.20 | 0.00 |
| **LSTM^1^** | - | - | 0.03 | 0.04 | **9.20** | 1.22 | 2.45 | 1.31 | **9.86** | 3.79 | 0.05 | 0.80 | 1.68 | **4.67** | 0.39 |
| **LSTM^2^** | - | - | - | 0.00 | **12.06** | 1.14 | 2.8 | 2.09 | **11.73** | 3.17 | 0.21 | 1.94 | 2.14 | **6.18** | 0.87 |
| **CNN** | - | - | - | - | **9.23** | 0.57 | 2.26 | 1.67 | **6.60** | 2.52 | 0.19 | 0.99 | 1.75 | **4.78** | 0.61 |
| **CNN^1^** | - | - | - | - | - | **19.71** | 2.22 | **4.24** | **30.45** | **19.53** | **7.78** | **5.72** | 2.76 | 0.86 | **5.57** |
| **CNN^2^** | - | - | - | - | - | - | **5.99** | **5.05** | **4.81** | 0.66 | 1.95 | **5.19** | **5.62** | **10.33** | 3.23 |
| **LSTM-CNN** | - | - | - |  | - | - | - | 0.04 | **19.35** | **11.21** | 1.52 | 0.33 | 0.02 | 0.34 | 0.52 |
| **LSTM-CNN^1^** | - | - | - | - | - | - | - | - | **15.76** | **7.95** | 0.72 | 0.09 | 0.00 | 0.80 | 0.20 |
| **LSTM-CNN^2^** | - | - | - | - | - | - | - | - | - | 1.29 | **10.84** | **18.09** | **17.64** | **24.37** | **14.77** |
| **RCNN** | - | - | - | - | - | - | - | - | - | - | **6.12** | **8.20** | **9.64** | **13.99** | **5.81** |
| **RCNN^1^** | - | - | - | - | - | - | - | - | - | - | - | 0.47 | 0.95 | 3.41 | 0.13 |
| **RCNN^2^** | - | - | - | - | - | - | - | - | - | - | - | - | 0.12 | 1.85 | 0.03 |
| **HieLSTM** | - | - | - | - | - | - | - | - | - | - | - | - | - | 0.91 | 0.36 |
| **HieLSTM^1^** | - | - | - | - | - | - | - | - | - | - | - | - | - | - | 3.39 |

^1^ denotes the model with the static PPI pre-trained module; ^2^ denotes the model with the tuned PPI pre-trained module. The bold values denote significant differences at the 0.05 level.

**References**

1. Bojanowski, P., Grave, E., Joulin, A., Mikolov, T. (2016) Enriching word vectors with subword information. *arXiv preprint arXiv:1607.04606*.

2. Mikolov, T., Sutskever, I., Chen, K., Corrado, G.S., Dean, J. (2013) Distributed representations of words and phrases and their compositionality. *Advances in neural information processing systems*, pp. 3111-3119.

3. Dietterich, T.G. (1998) Approximate statistical tests for comparing supervised classification learning algorithms. *Neural computation*, **10**, 1895-1923.
